# Difference in virulence between *Neisseria meningitidis* serogroups W and Y in transgenic mice

**DOI:** 10.1186/s12866-020-01760-4

**Published:** 2020-04-15

**Authors:** Lorraine Eriksson, Bianca Stenmark, Ala-Eddine Deghmane, Sara Thulin Hedberg, Olof Säll, Hans Fredlund, Paula Mölling, Muhamed-Kheir Taha

**Affiliations:** 1 Department of Laboratory Medicine, Faculty of Medicine and Health, Örebro University, Örebro, Sweden; 2 Institut Pasteur, Invasive Infections Unit, Paris, France; 3 Department of Infectious Diseases, Faculty of Medicine and Health, Örebro University, Örebro, Sweden

**Keywords:** *Neisseria meningitidis*, Serogroup W, Serogroup Y, Transgenic mice, Virulence

## Abstract

**Background:**

*Neisseria meningitidis* serogroups W and Y are the most common serogroups causing invasive meningococcal disease in Sweden. The majority of cases are caused by the serogroup W UK 2013 strain of clonal complex (cc) 11, and subtype 1 of the serogroup Y, YI strain of cc23. In this study, virulence factors of several lineages within cc11 and cc23 were investigated in transgenic BALB/c mice expressing human transferrin.

Transgenic mice were infected intraperitoneally with serogroup W and Y isolates. Levels of bacteria and the proinflammatory cytokine CXCL1 were determined in blood collected 3 h and 24 h post-infection. Apoptosis was investigated in immune cells from peritoneal washes of infected mice. Adhesion and induction of apoptosis in human epithelial cells were also scored.

**Results:**

The levels of bacteraemia, CXCL1, and apoptosis were higher in serogroup W infected mice than in serogroup Y infected mice. Serogroup W isolates also induced higher levels of apoptosis and adhesion in human epithelial cells. No significant differences were observed between different lineages within cc11 and cc23.

**Conclusions:**

*N. meningitidis* Serogroup W displayed a higher virulence in vivo in transgenic mice, compared to serogroup Y. This was reflected by higher bacteremia, proinflammatory activity, and ability to induce apoptosis in mouse immune cells and human epithelial cells.

## Background

*Neisseria meningitidis* is a human pathogen that can cause invasive meningococcal disease (IMD), dominated by septicaemia and meningitis, but can also be carried asymptomatically in the throat and nasopharynx [1]. *N. meningitidis* is classified into serogroups, which are differently distributed worldwide [1, 2]. The bacteria can be further classified into sequence types (ST) by multilocus sequence typing (MLST), where genetically related isolates are grouped into clonal complexes (ccs), which can consist of different but closely related STs [3]. Invasive isolates usually belong to a few cc, known as hyperinvasive cc. Invasive *N. meningitidis* isolates have been shown to induce apoptosis in epithelial cells [4]. This induction seems to involve several bacterial structures such as the IgA protease, the outer membrane protein porin B (PorB), and the lipooligosaccharide (LOS) [5–7].

*N. meningitidis* serogroups W (NmW) and Y (NmY) are currently the major serogroups causing IMD in Sweden, with incidences (and proportions) of 0.22 (44%) and 0.12 (22%) per 100,000 population respectively in 2018. The incidence of NmY has been high in Sweden since 2005, with the increase due to the YI strain of cc23 [8–10]. YI consists of two genetically distinct subtypes, one of which (subtype 1) has been determined as the cause of the increased incidence [9]. An increase in NmY has also been reported from other European countries [11]. The increased incidence of NmW is due to strains that are similar to the NmW UK 2013 strain of cc11 [12]. This strain belongs to the NmW South American/UK sub-lineage of cc11. The sub-lineage consists of three strains: the South American strain, the original UK strain, and the UK 2013 strain (hereafter called the 2013 strain) [13, 14]. An increase in NmW has been reported from several European countries [14–17], and in 2015, the 2013 strain was involved in an outbreak at an international scout jamboree in Japan, which resulted in invasive cases reported from Scotland and Sweden [13].

The genetic differences within YI cc23 and the South American/UK sub-lineage of cc11 are few, and cannot explain the observed difference in incidence of the subtypes or strains in these serogroups [9, 12]. However, the impact of these differences on the bacterial virulence has not been explored experimentally. A transgenic BALB/c mouse model that expresses human transferrin can be used as a reliable tool to study meningococcal virulence, as it provides bacteria with a human source of iron that is required for bacterial growth. This model allows consistent bacterial growth in blood of infected mice, since meningococci can acquire iron from human transferrin as seen during invasive infection in humans [18, 19]. The aim of this study was to investigate virulence factors using an experimental pathophysiological comparison between and within NmW and NmY isolates that gives an increased incidence of IMD in a defined system using a transgenic mouse model expressing human transferrin.

## Results

### Survival of meningococcal isolates in transgenic mice

Transgenic BALB/c mice expressing human transferrin were infected with NmY and NmW isolates. The NmY isolates belonged to YI subtype 1 (*n* = 6), YI subtype 2 (*n* = 3), and cc23 but not YI (*n* = 1, called cc23 other). The NmW isolates belonged to the original UK strain (*n* = 3) and the 2013 strain (*n* = 10). Four of the 2013 strain isolates were connected to the 2015 World Scout Jamboree in Japan, one was invasive, and the other three were nasopharynx/throat isolates collected from close contacts during the outbreak.

**Fig. 1:**
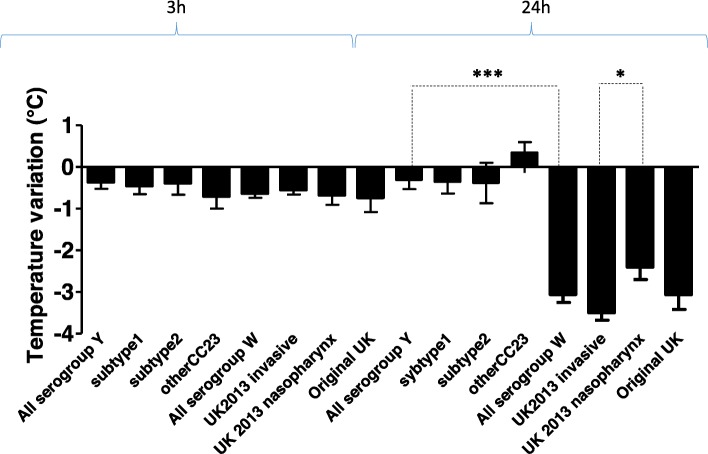
Temperature variation in mice infected with *Neisseria meningitidis* serogroup W and Y isolates. Temperature was measured transcutaneously using an infrared device 3 h and 24 h post- infection and expressed as differences in °C from the temperature before infection for each individual mouse in each group as indicated. The mean differences and standard errors are shown for each group. *** indicates the level of the significant difference in temperature between mice infected with serogroup W isolates compared to serogroup Y infected mice (*p* < 0.0001). * indicates the level of the significant difference between mice infected with the invasive serogroup W UK 2013 strain compared to mice infected with the UK 2013 strain isolates from the nasopharynx/throat (*p* < 0.01)

Three hours post-infection, the mice showed slight hypothermia but there were no significant differences between the serogroups. After 24 h of infection, more pronounced hypothermia was observed in mice infected with NmW isolates, differing significantly from NmY infected mice, which showed no hypothermia (*p* < 0.0001). Moreover, mice infected with the NmW 2013 strain invasive isolates showed significantly more hypothermia than those infected with NmW 2013 strain isolates from the nasopharynx/throat (*p* < 0.01) (Fig. 1).

**Fig. 2:**
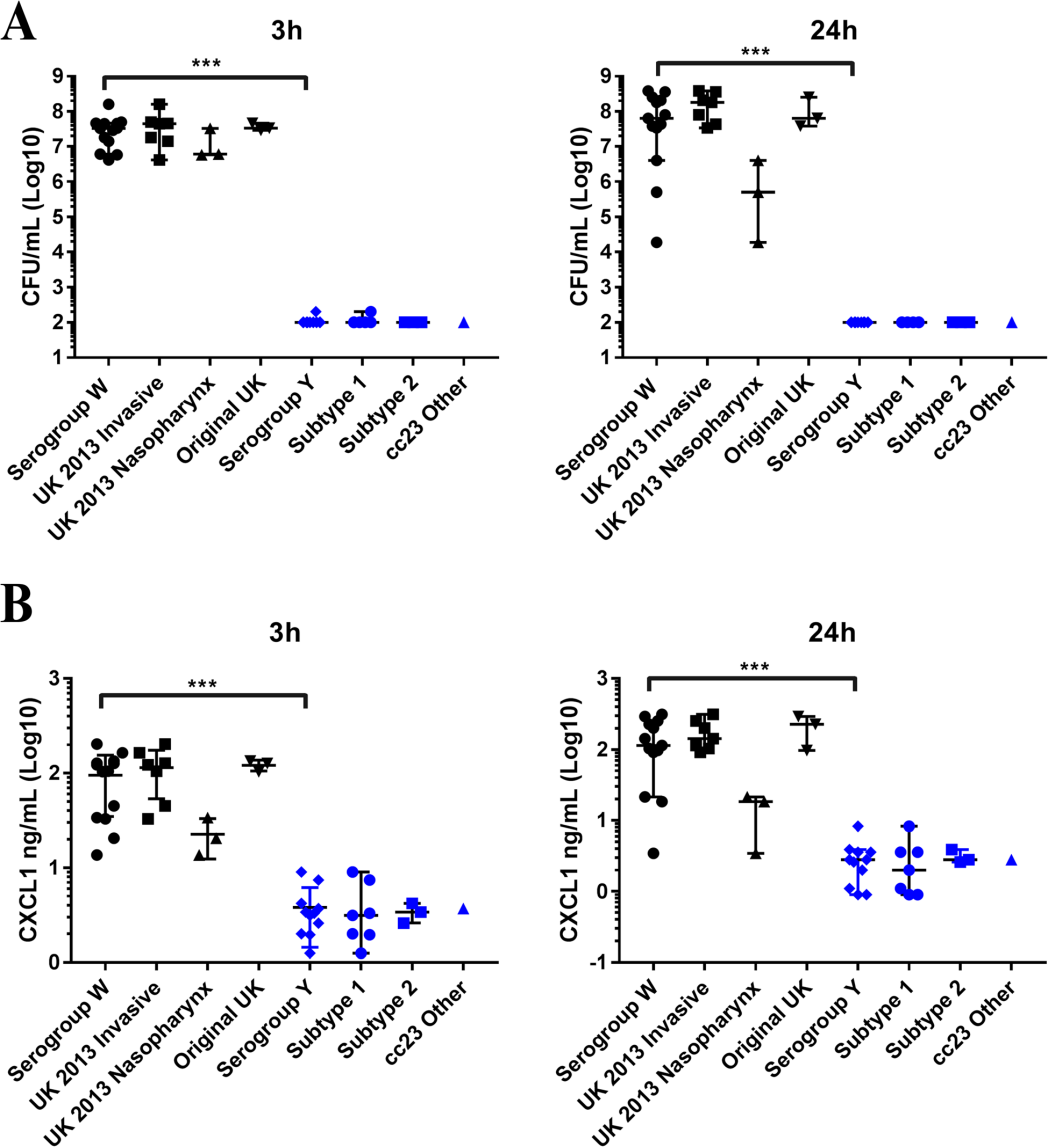
**a**: Amount of bacteria (CFU/mL) in blood 3 h and 24 h post-infection with *N*. *meningitidis* serogroup W and Y isolates. The bars represent the median and 95% confidence interval. Differences between and within serogroups were compared using a Mann-Whitney test. *** indicates the level of the significant difference between serogroup W infected mice compared to serogroup Y infected mice (*p* < 0.0001). **b**: Amount of the proinflammatory cytokine CXCL1 in ng/mL detected in blood of *N*. *meningitidis* serogroup W and Y infected mice after 3 h and 24 h. The bars represent the median and 95% confidence interval. Differences between and within serogroups were compared using a Mann-Whitney test. *** indicates the level of the significant difference between serogroup W infected mice compared to serogroup Y infected mice (*p* < 0.0001)

Three hours post-infection, bacteria were detectable in the blood of infected mice. Blood concentrations in terms of colony forming units (CFUs) per ml were significantly higher in NmW infected mice (*n* = 39) than in NmY infected mice (*n* = 30) after both 3 h and 24 h (*p* = < 0.0001) (Fig. 2a). The NmW infected mice had a higher mortality; none of the NmY infected mice died, whereas 15 (infected with two original UK and three 2013 strain invasive isolates) of the 39 NmW infected mice died (Table 1). NmW infected mice had a significant increase in the amount of bacteria after 24 h compared to 3 h (*p* = 0.0105).

**Table 1:** Bacterial and clinical data for the *Neisseria meningitidis* isolates

PubMLST ID	Serogroup	Clonal complex (cc)	Sequence type (ST)	Subtype/ strain	Collection year	Diagnosis/ infection focus	Main symptoms other than fever	Patient died < 30 days	Mice died < 24 h
57572	W	cc11	1287	Original UK strain	2011	Sepsis, pneumonia	Gastrointestinal	No	Yes
57573	W	cc11	11	Original UK strain	2012	Septic chock	Gastrointestinal	Yes	No
57582	W	cc11	11	Original UK strain	2016	Bacteraemia, pneumonia	Respiratory (cough)	No	Yes
57574	W	cc11	11	2013 strain	2014	Bacteraemia, epiglottitis	Respiratory (sore throat)	No	Yes
57575	W	cc11	11	2013 strain	2014	Bacteraemia, tonsillitis	Respiratory (sore throat)	No	Yes
57576	W	cc11	11	2013 strain	2015	Bacteraemia, epiglottitis	Respiratory (sore throat)	No	No
57581	W	cc11	11	2013 strain	2016	Bacteraemia, no focus	Back pain, myalgia	No	Yes
57583	W	cc11	11	2013 strain	2016	Bacteraemia, pneumonia	Gastrointestinal	Yes	No
57584	W	cc11	11	2013 strain	2016	ND	ND	ND	No
57577	W	cc11	11	2013 strain	2015	Meningitis, bacteraemia,	Back pain, myalgia	No	No
57578	W	cc11	11	2013 strain	2015	^a^	^a^	^a^	No
57580	W	cc11	11	2013 strain	2015	^a^	^a^	^a^	No
57579	W	cc11	11	2013 strain	2015	^a^	^a^	^a^	No
41339	Y	cc23	23	YI Subtype 1	2006	Bacteraemia, meningitis	Respiratory (sore throat), gastrointestinal	No	No
41341	Y	cc23	23	YI Subtype 1	2011	Pneumonia, bacteraemia	Lowered general condition	No	No
51890	Y	cc23	23	YI Subtype 1	2013	ND	ND	ND	No
41343	Y	cc23	23	YI Subtype 1	2012	Bacteraemia, meningitis	Tiredness	No	No
41344	Y	cc23	23	YI Subtype 1	2012	Bacteraemia, meningitis	Headache	No	No
41342	Y	cc23	23	YI Subtype 1	2012	Meningitis	Headache, gastrointestinal	No	No
41340	Y	cc23	23	YI Subtype 2	2011	Bacteraemia, meningitis	Gastrointestinal, myalgia	No	No
41337	Y	cc23	23	YI Subtype 2	1995	ND	ND	ND	No
41338	Y	cc23	23	YI Subtype 2	1998	Meningitis	Gastrointestinal, headache	No	No
41611	Y	cc23	4183	cc23 other	2009	Bacteraemia, meningitis	Headache, gastrointestinal, respiratory	No	No

ND No data available for this patient

^a^Nasopharynx/throat isolates collected from close contacts during an outbreak

### Induction of the proinflammatory cytokine CXCL1

The blood collected after infection in mice was also used to investigate levels of the proinflammatory cytokine CXCL1. At both 3 h and 24 h, NmW infected mice showed significantly higher levels of CXCL1 than NmY infected mice (*p* < 0.0001, Fig. 2b). No statistical difference was observed between the strains or subtypes of NmW and NmY.

### Induction of apoptosis

**Fig. 3:**
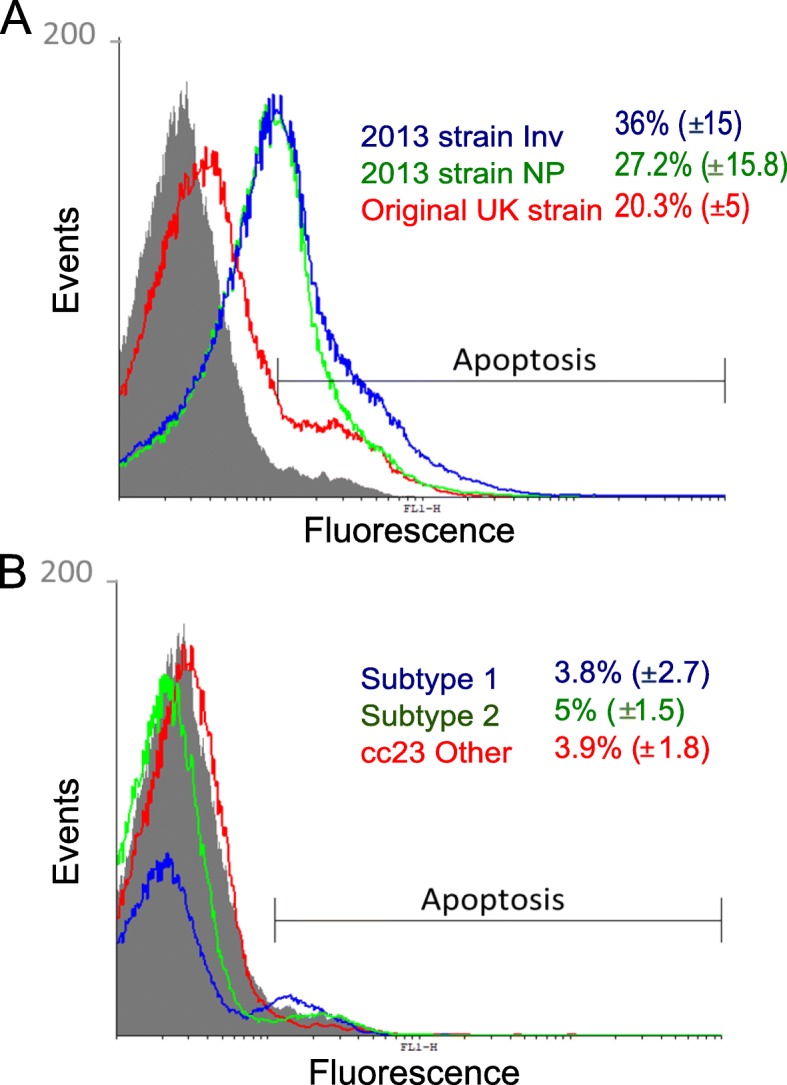
Histogram overlays of apoptosis of immune cells in the peritoneal washes of infected mice. The apoptosis is displayed as fluorescence levels on the x-axis. Three isolates represent each lineageof the *N*. *meningitidis* serogroups. The data from infected mice were compared to data from two non-infected mice as controls. Serogroup W isolates are displayed in histogram **a:** non-infected mice (grey), original UK isolate (red), invasive 2013 strain isolate (blue), and nasopharynx/throat (NP) 2013 strain isolate (green). Serogroup Y isolates are displayed in histogram **b**: YI subtype 1 (blue), YI subtype 2 (green), cc23 other (red), and non-infected mice (grey). The percentages of cells in apoptosis and standard errors are displayed for each isolate as indicated by the corresponding colours

Apoptosis of mouse immune cells was investigated in mice infected with one isolate from each strain or subtype of NmW and NmY. Within their respective groups, strains of NmW and the subtypes of NmY did not differ much in terms of the percentage of apoptosis in mouse immune cells recovered from the peritoneal washes (Fig. 3a). However, all NmY isolates induced lower levels of apoptosis than NmW isolates (Fig. 3b).

**Fig. 4:**
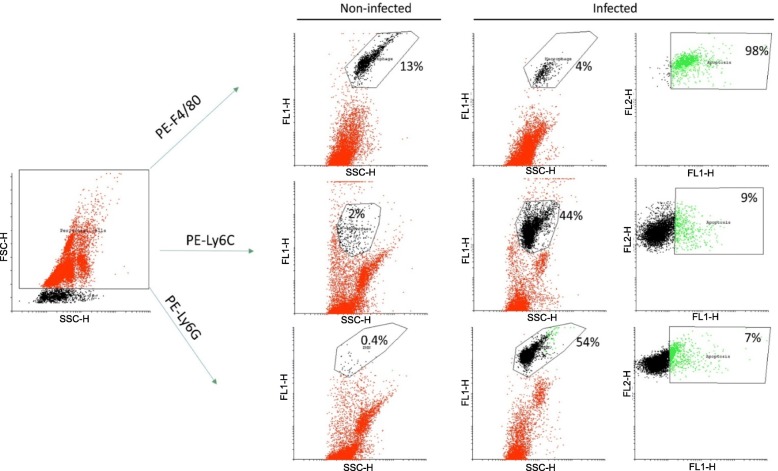
Staining of immune cells in peritoneal wash from one representative *N*. *meningitidis* serogroup W (isolate PubMLST ID 57576) infected mouse, 3 h post-infection compared to a non-infected mouse. The defined immune cells are gated on the forward (FSC) and scattered (SSC) axis and are seen in red. These cells were then observed according to the specific phycoerythrin (PE) fluorochrome staining (FL2 axis) for macrophages (F4/80+), monocytes (Ly6C+), and neutrophils (Ly6G+), represented in black for both infected and non-infected mice. In the infected mouse, these cells were gated for apoptosis using FITC-labelled Annexin V (FL1 axis), which is represented by green colour. The percentage was calculated for gated cells from all the observed cells, which is indicated in each gate as well as the names of gated cells

One mouse infected with the NmW invasive 2013 strain isolate, PubMLST ID 57576 (P1.5–2, 36–2, F1–1, ST-11) was compared to a non-infected mouse in order to gate the inflammatory cells. Specific fluorescence labelling was employed to detect neutrophils (Ly6G+), monocytes (Ly6C+), and macrophages (F4/80+) in the peritoneal washes. Large recruitments of neutrophils (Ly6G+) and monocytes (Ly6C+) were detected at the site of infection compared to a non-infected mouse. Moreover, low proportions of these cells underwent apoptosis (7 and 9%, respectively). Conversely, resident macrophages (F4/80+) were present in lower proportions in the infected mouse than in the non-infected mouse (4 and 13%, respectively). This reduction was correlated with a high level of induction of apoptosis in this macrophage population (98%) (Fig. 4).

### Interaction of meningococcal isolates with human epithelial cell line

**Fig. 5:**
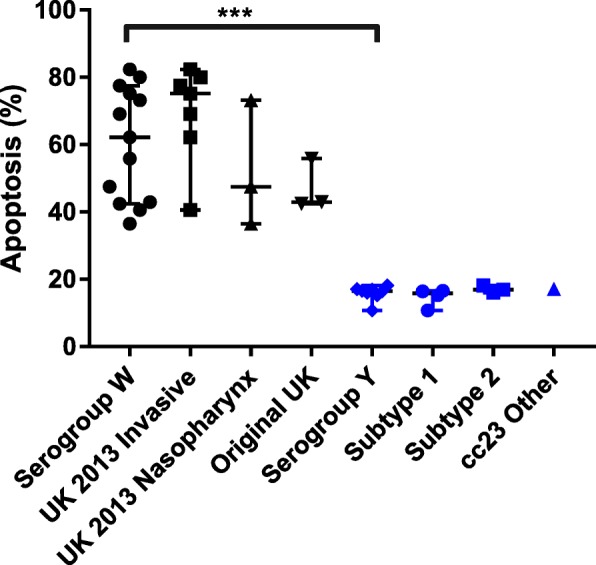
Percentage of apoptosis in Hec-1-B cells 3 h post-infection with serogroup *N. meningitidis* W and Y isolates. Lines represent the median and 95% confidence intervals. The comparison between and within serogroups was performed using a Mann-Whitney test. *** indicates the significant difference in apoptosis between mice infected with serogroup W isolates compared to serogroup Y infected mice (*p*<0.0001)

To correlate our results in mice to human infection at the primary site of infection (the epithelial cells), we compared the interaction of NmW and NmY isolates with human endometrial carcinoma epithelial cell line Hec-1-B cells. NmW infected cells induced a higher percentage of apoptosis (*p* < 0.0001) (Fig. 5). However, there was no significant difference between the different lineages within NmW and NmY.

**Fig. 6:**
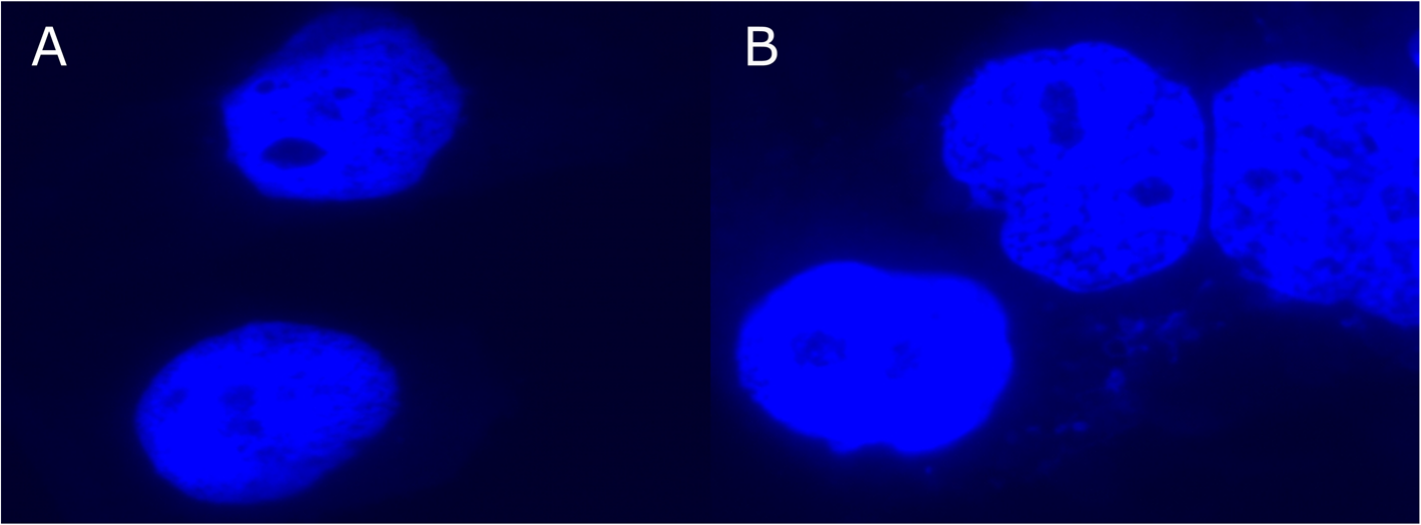
DAPI staining of Hec-1-B cells. **a:** Hec-1-B cells infected with a *N*. *meningitidis* serogroup Y isolate without adherent bacteria, **b:** Hec-1-B cells infected with a *N*. *meningitidis* serogroup W isolate with adherent bacteria as revealed by DAPI (which stains nucleic acids)

**Fig. 7:**
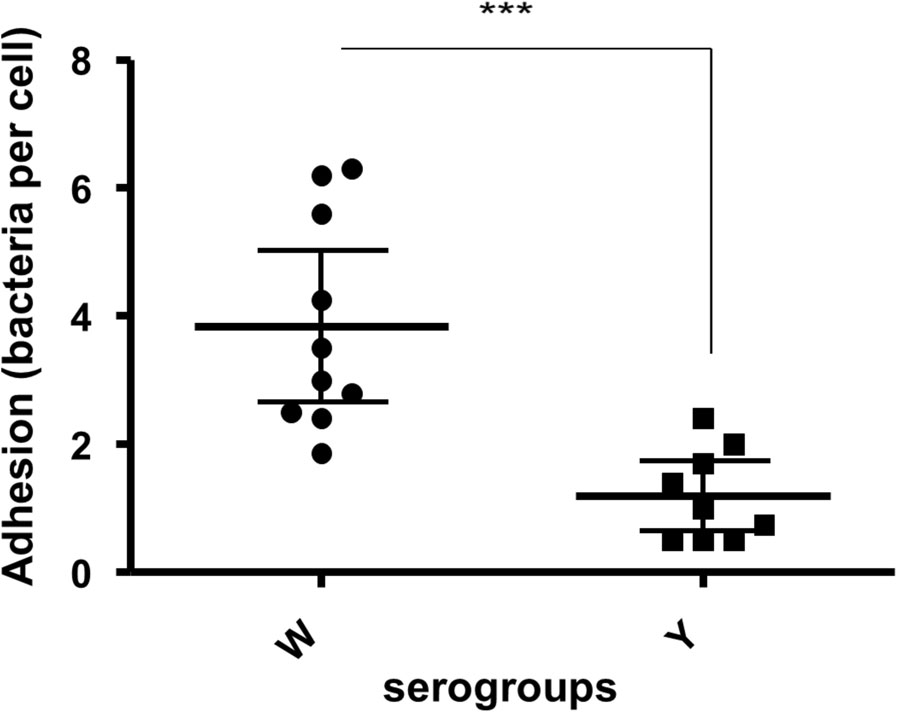
Comparison of number of bacteria adherent to cells 3 h post-infection. Lines represent the mean and 95% confidence intervals. A Mann-Whitney test was used to compare serogroups W and Y. *** indicates the level of the significant difference in adhesion between mice infected with serogroup W isolates compared to serogroup Y infected mice (*p* = 0.0004)

NmY infected cells had few adherent bacteria whilst NmW infected cells had higher amounts of adherent bacteria (Fig. 6). The difference in adherent bacteria was statistically significant (*p* = 0.0004) (Fig. 7). There was no difference in adherence between lineages in the serogroups.

### Genomic analysis and allelic differences in genes involved in apoptosis, inflammatory response, LOS, and iron acquisition

Whole genome sequencing (WGS) data from the NmW and NmY isolates were used to analyse and compare specific genes known to be involved in inflammatory response and induction of apoptosis. The most frequent alleles of *iga*, *porB, ponA, penA,* genes involved in iron acquisition (*lbpA*, *lbpB*, *tbpA*, *tbpB*, *hpuA*, *hpuB*, *hmbR*), and genes encoding LOS (*mshA*, *rlpB*, *rfaF*, *lgtF*, *rfaK*, *YhbG*, *lptC*, *lgtE*, *lgtB*, *lpt3*, *lpt6* and *rfaC*) are displayed in Table 2. The frequency of these alleles was also examined among all NmW (*n* = 3040) and NmY (*n* = 1590) isolates present in PubMLST (Table 2). The number of amino acids that differed between the most frequent alleles found in the NmW and NmY isolates are also given in Table 2. All NmW isolates had PorB2, and all NmY YI isolates had PorB3 except NmY cc23 other, which had PorB2.

**Table 2:** Allelic and amino acid difference in genes involved in apoptosis, iron acquisition, and LOS between *Neisseriameningitidis* serogroup W and Y isolates

Locus	Gene name	Gene product	Function	Allele Serogroup Y (frequency)	Allele Serogroup W (frequency)	Difference in amino acids between serogroups W and Y	Frequency of alleles in isolates in PubMLST
NEIS2020	*porB*	Porin, major outer membrane protein	Inflammatory response/ induction of apoptosis	67 (8/10)	244 (12/13)	142 aa	Y: 37% W: 47%
NEIS0651	*iga*	IgA1 protease	Inflammatory response/ induction of apoptosis	189 (9/10)	37 (10/13)	72 aa	Y: 60% W: 74%
NEIS0414	*ponA*	Penicillin-binding protein 1	Inflammatory response/ induction of apoptosis	45 (9/10)	1 (10/13)	2 aa	Y: 71% W: 95%
NEIS1753	*penA*	Penicillin-binding protein 2	Inflammatory response/ induction of apoptosis	8 (10/10)	59 (11/13)	0 aa	Y: 70% W: 74%
NEIS1468	*lbpA*	Lactoferrin binding protein A	Iron acquisition	226 (9/10)	8 (9/13)	33 aa	Y: 51% W: 82%
NEIS1469	*lbpB*	Lactoferrin binding protein B	Iron acquisition	137 (9/10)	5 (7/13)	155 aa	Y: 52% W: 49%
NEIS1690	*tbpA*	Transferrin binding protein A	Iron acquisition	1054 (3/10)	2 (11/13)	221 aa	Y: 3.8% W: 89%
NEIS1691	*tbpB*	Transferrin binding protein B	Iron acquisition	1111 (3/10)	1 (12/13)	441 aa	Y: 1.4% W: 81%
NEIS1946	*hpuA*	Haemoglobin-haptoglobin utilisation protein	Iron acquisition	110 (4/10)	776 (3/13) Absent (9/13)	70 aa	Y: 44% W: 52% (Absent), 13% (776)
NEIS1947	*hpuB*	Haemoglobin-haptoglobin utilisation protein	Iron acquisition	35 (10/10)	1898 (9/13)	39 aa	Y: 67% W: 32%
hmbR	*hmbR*	Haemoglobin receptor protein	Iron acquisition	Absent (10/10)	8 (6/13) 9 (6/13)	N/A	Y: 98% W: 21% (8), 46% (9)
NEIS0304	*mshA*	Lipid A export ATP-binding/permease protein (IM)	LOS	118 (9/10)	89 (12/13)	2 aa	Y: 82% W: 28%
NEIS0657	*rlpB*		LOS	3 (9/10)	1 (12/13)	0 aa	Y: 75% W: 95%
NEIS1456	*rfaF*	Heptosyltransferase II	LOS	22 (10/10)	4 (13/13)	6 aa	Y: 77% W: 71%
NEIS1618	*lgtF*	Beta-1,4 glucosyltransferase	LOS	57 (9/10)	43 (13/13)	2 aa	Y: 70% W: 80%
NEIS1619	*rfaK*	Alpha-1,2 N-acetylglucosamine transferase	LOS	31 (10/10)	92 (12/13)	12 aa	Y: 77% W: 52%
NEIS1812	*yhbG*	Lipopolysaccharide ABC transporter	LOS	107 (6/10)	1 (12/13)	12 aa	Y: 9.4% W: 88%
NEIS1814	*lptC*	Lipopolysaccharide export system protein (OM)	LOS	2 (10/10)	1 (12/13)	3 aa	Y: 89% W: 86%
NEIS1900	*lgtE*	Lacto-N-neotetraose biosynthesis glycosyl transferase	LOS	162;206 (6/10)	45;321 (13/13)	101 aa	Y: 40% W: 52%
NEIS1901	*lgtB*	Lacto-N-neotetraose biosynthesis glycosyl transferase	LOS	81 (6/10)	79 (13/13)	10 aa	Y: 61% W: 56%
NEIS1986	*lpt3*	LOS O-3 PEA transferase	LOS	38 (1/10) Absent (9/10)	4 (12/13)	13 aa	Y: 7.7% (38), 51% (Absent) W: 80%
NEIS2012	*lpt6*	O-6 PEA LOS transferase	LOS	Absent (10/10)	3 (13/13)	N/A	Y: 96% W: 84%
NEIS2134	*rfaC*	Heptosyltransferase I	LOS	3 (10/10)	1 (13/13)	17 aa	Y: 82% W: 60%

aa Amino acids

N/A Not applicable

LOS Lipooligosaccharide

W *Neisseria meningitidis* Serogroup W

Y *Neisseria meningitidis* Serogroup Y

**Fig. 8:**
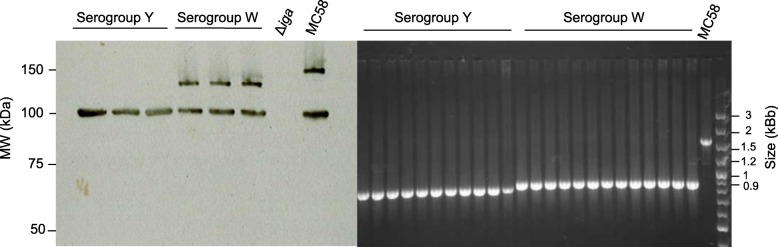
**a**: Western blot of IgA1 protease in a collection of serogroup W and Y isolates and compared to a serogroup B isolate (MC58). A mutant strain, *Δiga*, was used as a negative control [6]. The IgA1 protease consisting of the IgaP and Igaα domains generates a protein of size 150 kDa in MC58. An IgA1 protease consisting of only the IgaP domain results in a protein with a size of 100 kDa in MC58. The serogroup W isolates generated a protein that was smaller than 150 kDa. **b**: PCR amplified Igaα domains of serogroup W and Y isolates compared to MC58, which has a complete Igaα domain. The complete Igaα domain of MC58 has a size of approximately 1.5 kb while the serogroup W and Y isolates are smaller, approximately 0.9 kb. The serogroup Y isolates seem to have a slightly smaller Igaα domain than serogroup W

Isolates were investigated for the IgA1 protease (IgaP and Igaα domains) as well as the IgaP domain alone using western blotting and PCR. The whole IgA1 protease (IgaP and Igaα domains) was present in all the NmW isolates and none of the NmY isolates, as identified by western blot (Fig. 8a). However, the IgaP domain was present in both NmW and NmY (Fig. 8a). The Igaα domains of NmW and NmY isolates were compared to a serogroup B isolate (MC58) by PCR. Serogroups W and Y both had smaller Igaα domains than MC58 (Fig. 8b).

Allelic differences in LOS encoding genes between NmW and NmY isolates are shown in Table 2. One locus (*lpt6*) was present in all of the NmW isolates and none of the NmY isolates; this encodes O-6 PEA LOS transferase that adds phosphoethanolamine (PEA) to the LOS (Table 2). When all NmY isolates (*n* = 1590) in PubMLST were checked, this gene was absent in 96% of the isolates. The difference in amino acids in LOS genes due to differences in the most common alleles of NmW and NmY is displayed in Table 2.

*N. meningitidis* has several proteins for iron acquisition, including lactoferrin binding proteins A and B (NEIS1468, NEIS1469), transferrin binding proteins A and B (NEIS1693, NEIS1691), haemoglobin-haptoglobin utilisation proteins (NEIS1946, NEIS1947), and haemoglobin receptor (*hmbR*). Genes were compared for the NmW and NmY isolates and the difference in amino acids in these genes in displayed in Table 2. The gene coding for HmbR was missing in the NmY isolates, while it was present in the NmW isolates. When PubMLST was checked, this gene was missing in 98% of the NmY isolates.

## Discussion

In this study, we propose an approach that allows global comparison of virulence between isolates in close linkage to genotypic characterisation of the isolates. This approach measures the ability to provoke invasive infection in mice and its correlation with the induction of apoptosis at the site of infection as well as the induction of systemic inflammatory response. Using transgenic mice expressing the human transferrin, we were able to show that NmW isolates were more invasive, were able to provoke death of mice, and induced higher levels of the proinflammatory cytokine CXCL1, when compared to NmY isolates. We used the intraperitoneal route of infection, which in contrast to the respiratory route is not the natural route of invasive meningococcal infections [20, 21] However, the intraperitoneal route in this animal model allows more consistent blood invasion and therefore allows analysis of virulence in terms of ability to grow in blood and spread. Although there were significant differences between the serogroups, no significant differences could be detected among the strains or subtypes within each serogroup.

The NmW and NmY isolates used in this study caused both respiratory and gastrointestinal symptoms in humans, with diagnoses and infection foci ranging from tonsillitis and epiglottis to sepsis and meningitis (Table 1). There was, however, no clear connection between the severity of disease in patients and mortality observed in mice. Two of the patients, infected with the original UK and 2013 strains, died within 30 days. Conversely, the mice infected with these isolates did not die within 24 h of infection.

The NmW invasiveness was associated with higher induction of apoptosis in the inflammatory cells from the peritoneal washes (Figs. 3 and 4). By inducing apoptosis in macrophages and other resident immune cells, bacteria can gain an advantage in survival and spread within the host [22]. The higher levels of apoptosis in human epithelial cells infected with NmW isolates in comparison to NmY isolates, as well as higher levels of infection, are in agreement with this hypothesis.

We also investigated genes encoding PorB, penicillin binding protein 1 (PBP1), penicillin binding protein 2 (PBP2), LOS, and IgA1, as these structures can be sensed by host cells as danger signals and may influence the induction of apoptosis. A comparison of the most common alleles within these genes in NmW and NmY showed that the allelic differences between these serogroups caused changes in several amino acids in each gene (Table 2). All NmW had PorB2; this PorB type has been suggested to be more invasive in previous studies in rats [23, 24], which is in line with our finding of NmW being more invasive.

Our data suggest that iron acquisition systems differ between the NmY and NmW isolates in this sudy (Table 2). This may be linked to the different virulence observed in our mouse model, because the ability to use one of the systems of iron acquisition (the human transferrin) is scored. Interestingly, the haemoglobin receptor protein (HmbR) which has been reported to be linked with enhanced meningococcal virulence [25], was missing in NmY isolates but present in NmW. This gene was also missing in 98% of all NmY isolates in PubMLST. The difference in acquisition of iron by NmW isolates could enable better growth in the blood and in deep organs such as the spleen. However, meningococcal virulence relies on several virulence factors in addition to iron acquisition.

Invasive cc11 isolates have been connected to a higher ability to induce apoptosis. This is in part due to differences in IgA1 protease [5–7], which can induce apoptosis by entering the nucleus of host cells and interacting with NF-κB [6, 7]. Deletion in the *iga* gene can affect the size of the produced protein. If the bacteria is producing only the IgaP domain and not the whole IgA1 protease (IgaP and Igaα domains), it will not be able to induce apoptosis [6]. Our results (Fig. 8) from IgA PCR and Western blotting suggest that the *iga* alleles harboured by NmW isolates encode a protein with the nuclear transport signals that seem to be absent in the *iga* alleles of NmY isolates. Subsequently the IgA protease function differs between the two types of isolates. The role of the nuclear transport signal in inducing apoptosis has already been suggested [6]. We also observed a higher adhesion by NmW isolates on epithelial cells, which might allow more efficient delivery of these structures and hence contribute to the difference in virulence compared to NmY in this study, since adhesion to epithelial cells is the initial step required for colonization and invasion into the bloodstream [20, 21].

NmY isolates seem to be associated with initial respiratory infections in elderly people where other comorbidities can contribute to the outcome of IMD. Exploring the virulence of these NmY isolates may require the use of the respiratory route of infection in mice. In the present study, the *lpt6* gene encoding O-6 PEA LOS transferase that adds PEA to LOS was absent from the NmY isolates (Table 2). PEA is recognized by the immune system, leading to bacterial elimination and opsonophagocytosis in the infected host. The absence of O-6 PEA on the NmY isolates may mean that this phagocyte-mediated clearance of bacteria in the respiratory tract is avoided [26]. In line with our findings, 96% of the NmY isolates in PubMLST lacked this gene.

In future studies, genome-wide association will be performed to investigate differences in genes and how this affects the virulence of the bacteria in a larger collection of NmW and NmY isolates, as well as other serogroups of *Neisseriameningitidis*.

A limitation of this study is that only a few isolates from each lineage of NmW and NmY were included. It is possible that examination of additional isolates in each lineage would make it possible to observe statistically significant differences within the serogroups. In this study, we present allelic differences between the investigated isolates. However, these genetic differences have not been experimentally confirmed, which is needed in order to understand how they affect the phenotype.

## Conclusions

This study of NmW and NmY isolates found that the NmW isolates displayed high virulence in vivo in transgenic mice expressing human transferrin. The low levels of infection and apoptosis seen in NmY do not reflect the number of IMD cases due to this serogroup in Sweden. This study could not explain why specific lineages within these serogroups had a higher incidence than others, and further experiments are required to investigate whether there is a difference in virulence within these serogroups.

## Methods

### Bacterial isolates

Clinical isolates of *N. meningitidis* serogroups W (*n* = 13) and Y (*n* = 10) collected in Sweden were included in the study (Table 1). Ten of the NmW isolates belonged to the 2013 strain of cc11, including four isolates connected to the outbreak at the 2015 World Scout Jamboree in Japan (one invasive isolate and three nasopharynx/throat isolates collected from close contacts during the outbreak) [27]. Three invasive isolates belonging to the same linage as the original UK strain were also included (see Additional File 1). Ten NmY isolates of the YI strain of cc23 were included: six of subtype 1, three of subtype 2, and one that did not belong to a specific strain of cc23 (called cc23 other; see Additional File 2). Isolates were grown on gonococcal base agar (GCB) solid medium with Kellogg’s supplements [28] overnight at 37 °C with 5% CO_2_.

Information regarding diagnoses and mortality of patients infected with the NmW and NmY isolates in this study is displayed in Table 1. Three NmY and one NmW isolates did not have any patient information available.

### Infection in transgenic mice

The congenic BALB/c transgenic mice expressing human transferrin used in this study were established in the laboratory at the Pasteur Institut (using BALB/c mice from Janvier Labs, France) as previously described [19]. The mice were bred in-house and were kept in a biosafety containment facility in filter-topped cages with sterile litter, water, and food, according to institutional guidelines. This study used transgenic female BALB/c mice with an age of between 8 and 10 weeks and mean weight of 21.7 g. The mice were infected by intraperitoneal injections of 0.5 mL NmW and NmY bacterial suspensions of 5 × 10^7^ CFU/mL. No anaesthesia was used for intraperitoneal injection, as one intraperitoneal injection was considered in our protocol as a low-pain procedure, and we wished to avoid both additional injection and any potential effects on the bacterial infection. Each mouse was infected with one bacterial isolate, and each isolate was used in three separate experiments, requiring a total of 69 mice for the 23 isolates. Blood was drawn from the retro-orbital plexus of infected mice 3 h and 24 h post-infection in the laboratory. The blood was then plated, and bacterial count was determined after overnight incubation at 37 °C with 5% CO_2_. Cutaneous temperature was measured using an infrared thermometer (Bioseb) before infection and at 3 h and 24 h post-infection, and used to follow the clinical status of the mice during infection, as hypothermia has been reported as a sign of severe meningococcal infection in the mouse model [29]. Results were expressed as mean temperature loss in comparison with temperature before infection.

The experiment design in terms of mortality was reviewed and approved by the Institut Pasteur ethics committee. Our protocol stated that the study would be discontinued/interrupted if weight loss of more than 20% (not reached) or experimental death of the mouse due to infection occurred. The experiments were conducted for 24 h, at which point blood was drawn for the last time and all animals were euthanized. Health status and overall behaviour of the mice was checked every 6–8 h. It was important for the experiment that the animals were alive for the last blood draw, since blood was only drawn at two time points and a comparison of bacterial load would not have been possible if the animals had been euthanized earlier. Mice were euthanized by cervical dislocation or by intramuscular injection of high dose of a mixture of 10 mg/kg xylazine (Bayer, Puteaux, France) and 5 mg/kg ketamine (Merial, Lyon, France) according to the reviewed protocol. Death was verified when no heartbeat was detected. When mice were euthanized by injection, a cervical dislocation was also performed to ensure that the mice were dead.

### Determination of cytokine (CXCL1) levels

Post-infection with NmW and NmY isolates, blood was collected for analysis of cytokines. The proinflammatory cytokine CXCL1 was chosen as it has previously been shown to clearly and directly reflect the proinflammatory response in this model of systemic meningococcal infection [29]. It is expressed by inflammatory cells such as neutrophils and macrophages, and it has neutrophil chemotactic activity [30]. CXCL1 was measured by ELISA with the Mouse CXCL1/KC kit (R&D Systems, Minneapolis, MN, US), according to manufacturer’s instructions, in serum collected in the laboratory from mice 3 h and 24 h post-infection with NmW and NmY isolates. Samples were diluted as previously described [31].

### Induction of apoptosis in mice at the site of infection

Mice were infected as mentioned above using bacterial suspensions of 5 × 10^7^ CFU/mL with no anaesthesia. Experiments were conducted with one isolate from each lineage of NmW (*n* = 3) and NmY (n = 3). Each isolate was used to infect one mouse, and the experiments were repeated three times (total 18 mice). Three hours post-infection in the laboratory, mice were euthanatized by intramuscular injection of a mixture of 10 mg/kg Xylazine (Bayer) and 5 mg/kg ketamine (Merial). The peritoneal cavity was washed by injection and subsequent withdrawal of 2 mL saline. The cells in the peritoneal washes were stained using FITC-Annexin V (Enzo Life Sciences, Farmingdale, NY, USA) at 4 °C for 30 min in the dark, as previously described [5]. Apoptotic cells were gated for events showing fluorescent levels higher than the background (10 units on the FL1-H axis), which was determined using unstained cells.

As apoptosis was only observed in mice infected with NmW isolates (particularly the 2013 strain), we aimed to identify the type of inflammatory cells in the peritoneal washes. A separate experiment of flow cytometry with a FACSCalibur flow cytometer (BD Biosciences, San Jose, CA, USA) was performed as previously described [31]. In brief, we used markers for macrophages (F4/80-PE), neutrophils (Ly6G-PE), and monocytes (Ly6C-PE). Antibodies specific to mouse were purchased from BD Biosciences (Franklin Lakes, NJ, USA). Peritoneal washes of one mouse infected with NmW 2013 strain isolate and one non-infected mouse were performed post-infection in the laboratory. Mice were injected with a high dose of a mixture of 10 mg/kg xylazine (Bayer) and 5 mg/kg of ketamine (Merial) prior to the peritoneal wash.

### Adhesion to human Hec-1-B epithelial cells

The human endometrial carcinoma epithelial cell line Hec-1-B was used to evaluate meningococcal adhesion, as it represents a well established and frequently used model of adhesion [32]. In brief, Hec-1-B cells were cultured at 37 °C in a humidified atmosphere with 5% CO_2_ in RPMI 1640 supplemented with 10% heat-inactivated foetal bovine serum (FBS) (GIBCO, Waltham, MA, USA). Infection was carried out with ten NmW isolates and nine NmY isolates from each subgroup, at a multiplicity of infection (bacteria to cell ratio) of 10:1. Infection was performed by incubation at 37 °C for 3 h. After incubation, the cells were stained with DAPI (fluorescent blue nucleic acid staining) and observed under fluorescence microscope (ZEISS Microscopy, Jena, Germany). Viewing and counting of the bacteria was performed with the ZEN imaging software (ZEISS Microscopy). Adhesion was expressed as mean of number of bacteria per cell on the basis of counting 50 epithelial cells.

### Analysis and quantitative measurement of apoptosis in human Hec-1-B epithelial cells

Adhesion was conducted for 3 h as described above. The cells were stained using FITC-Annexin V and examined as above by flow cytometry with a FACSCalibur flow cytometer (BD Biosciences, USA).

### Differences in genes involved in apoptosis, inflammatory response, LOS, and iron acquisition

Previously published WGS data [33] available on the PubMLST *Neisseria * database (www.pubMLST.org/neisseria) was used to investigate isolates genetically with *N. meningitidis* cgMLST v1.0 [34]. Neighbour-net networks generated from the genomic comparisons were visualized using SplitsTree4 V4 (www.splitstree.org) [35] (Additional Files 1 and 2). WGS data were also analysed and compared for specific genes known to be involved in inflammatory response and induction of apoptosis using a “gene-by-gene” approach available through the PubMLST Genome Comparator tool of the BIGSdb. The investigated genes were *porB* (NEIS2020), *ponA* (NEIS0414), *penA* (NEIS1753), *iga* (NEIS0651), genes involved in iron acquisition (NEIS1468, NEIS1469, NEIS1693, NEIS1691, NEIS1946, and NEIS1947), and the genes encoding LOS (NEIS0304, NEIS0657, NEIS1456, NEIS1618, NEIS1619, NEIS1812, NEIS1814, NEIS1900, NEIS1901, NEIS1986, NEIS2012, and NEIS2134). The most frequent alleles of these genes were compared among all NmY (*n* = 1590) and NmW (*n* = 3040) isolates in PubMLST (accessed 4 March 2020).

### Western blotting and PCR of IgA1 protease

The IgA1 protease (IgaP and Igaα domains) and the IgaP domain alone were detected using rabbit polyclonal serum against the IgaP protease domain. The SDS-PAGE transfer to nitrocellulose was performed as previously described [6]. In brief, immunoreactive bands were visualized using appropriate HRP-conjugated secondary IgG antibody and ECL detection reagents (Amersham Pharmacia Biotech, GE Healthcare, Amersham, UK). A PCR amplifying the α-domain of IgA1 protease was performed as previously described [6] on a selection of NmW and NmY isolates.

### Statistical analysis

All experiments were repeated three times and an average calculated for each isolate. The NmW isolates were separated into the original UK strain, nasopharynx/throat isolates, and the invasive 2013 strain, and the NmY isolates into YI subtype 1, YI subtype 2, and cc23 other. The Mann-Whitney test was used to analyse differences between the serogroups in terms of CFU/ml and CXCL1 in mice as well as apoptosis and adhesion in cells. Apoptosis in mouse immune cells was analysed using a t-test. Values of *p* < 0.05 were considered statistically significant. When multiple comparisons were performed between isolates in the subtypes or strains of NmW and NmY, Bonferroni correction was used to correct the *p*-value. The Wilcoxon test was used to compare paired data within serogroups collected at different time points.

## Supplementary information

**Additional file 1.** Neighbour-net network of *Neisseria meningitidis* serogroup W isolates included in the study. Coloured dots represent different years. PubMLST ID is displayed for each isolate in the network. The nasopharynx/throat isolates of the 2013 strain are marked with a star (*). All isolates were clonal complex 11 except for isolate 57572, which was sequence type 1287.

**Additional file 2.** Neighbour-net network of *Neisseria meningitidis* serogroup Y isolates from Sweden included in the study. Coloured dots represent different years. PubMLST ID is displayed for each isolate in the network. All isolates were clonal complex 23 except isolate 41611, which was sequence type 4183.

## Data Availability

The genomic data for bacterial isolates analysed during the current study are available in the PubMLST *Neisseria* database, www.pubMLST.org/neisseria [33].

## Abbreviations

cc: Clonal complex
CFU: Colony forming unit
cgMLST: Core genome multilocus sequence typing
FBS: Fetal bovine serum
IMD: Invasive meningococcal disease
LOS: Lipooligosaccharide
MLST: Multilocus sequence typing
NmW: *Neisseria meningitidis* serogroup W
NmY: *Neisseria meningitidis* serogroup Y
PEA: Phosphoethanolamine
PBP1: Penicillin binding protein 1
PBP2: Penicillin binding protein 2
ST: Sequence type
WGS: Whole genome sequencing
