# Supplementary material for: Difference in virulence between Neisseria meningitidis serogroups W and Y in transgenic mice
Source: BMC Microbiol. 2020 Apr 15;20:92. doi: 10.1186/s12866-020-01760-4 (PMC7160935; doi:10.1186/s12866-020-01760-4)

Additional file 1. Neighbour-net network of Neisseria meningitidis serogroup W isolates included in the study. Coloured dots represent different years. PubMLST ID is displayed for each isolate in the network. The nasopharynx/throat isolates of the 2013 strain are marked with a star (*). All isolates were clonal complex 11 except for isolate 57572, which was sequence type 1287.

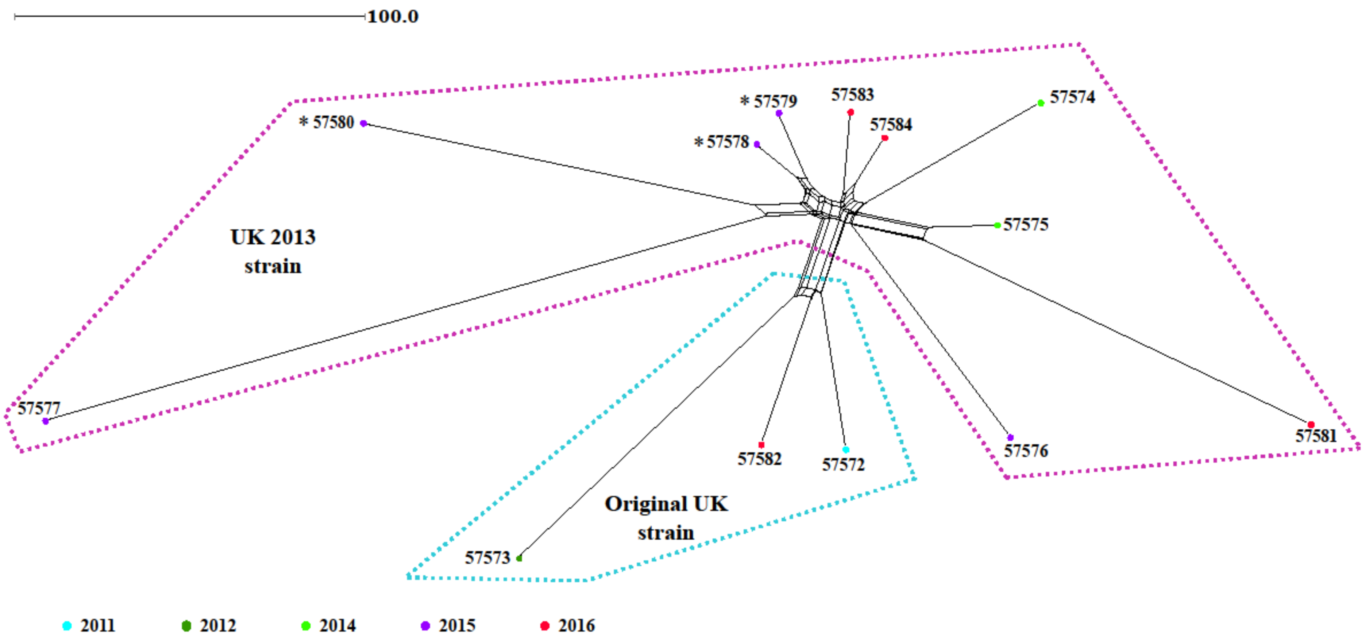

Supplement: Supplementary file 1 — Additional file 1. Neighbour-net network of Neisseria meningitidis serogroup W isolates included in the study. Coloured dots represent different years. PubMLST ID is displayed for each isolate in the network. The nasopharynx/throat isolates of the 2013 strain are marked with a star (*). All isolates were clonal complex 11 except for isolate 57572, which was sequence type 1287. [file 12866_2020_1760_MOESM1_ESM.pdf]
