# Supplementary material for: Difference in virulence between Neisseria meningitidis serogroups W and Y in transgenic mice
Source: BMC Microbiol. 2020 Apr 15;20:92. doi: 10.1186/s12866-020-01760-4 (PMC7160935; doi:10.1186/s12866-020-01760-4)

Additional file 2. Neighbour-net network of Neisseria meningitidis serogroup Y isolates from Sweden included in the study. Coloured dots represent different years. PubMLST ID is displayed for each isolate in the network. All isolates were clonal complex 23 except isolate 41611, which was sequence type 4183.

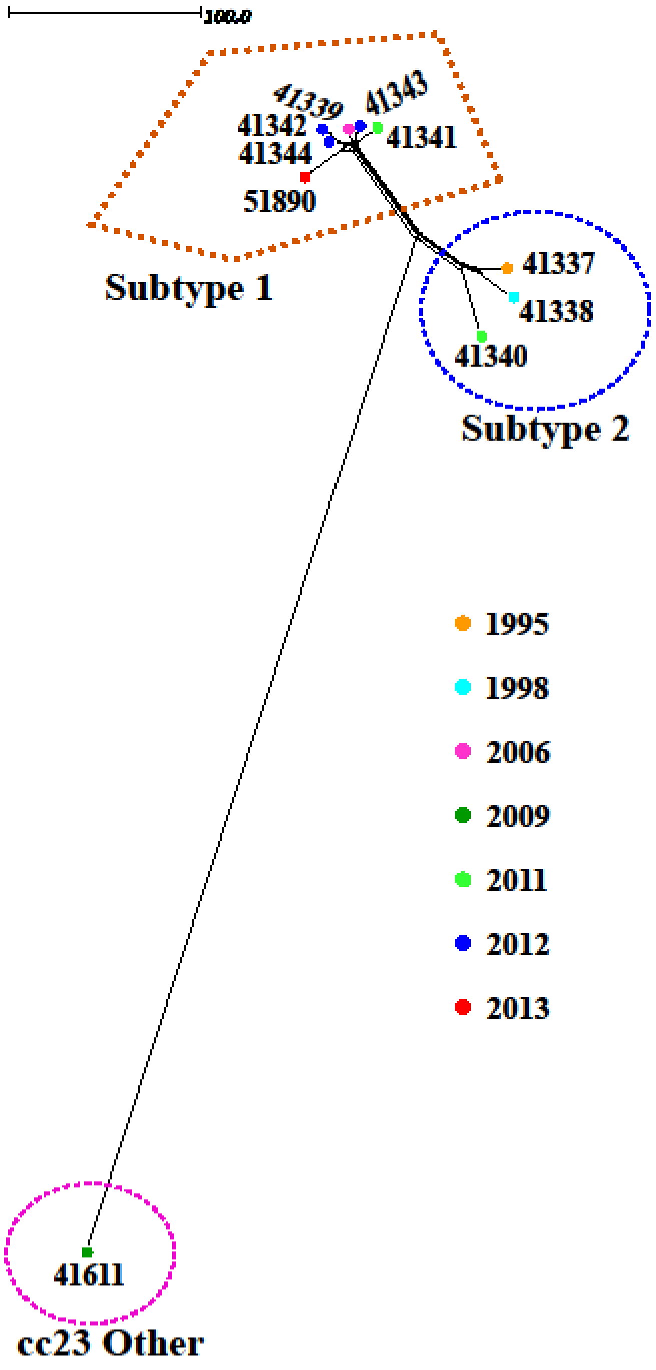

Supplement: Supplementary file 2 — Additional file 2. Neighbour-net network of Neisseria meningitidis serogroup Y isolates from Sweden included in the study. Coloured dots represent different years. PubMLST ID is displayed for each isolate in the network. All isolates were clonal complex 23 except isolate 41611, which was sequence type 4183. [file 12866_2020_1760_MOESM2_ESM.pdf]
